# Role of individual S4 segments in gating of Ca_v_3.1 T-type calcium channel by voltage

**DOI:** 10.1080/19336950.2018.1543520

**Published:** 2018-11-07

**Authors:** Bohumila Jurkovicova-Tarabova, Katarina Mackova, Lucia Moravcikova, Maria Karmazinova, Lubica Lacinova

**Affiliations:** a Center of Biosciences, Institute of Molecular Physiology and Genetics, Academy of Sciences, Bratislava, Slovakia; b Faculty of Natural Sciences, University of Ss. Cyril and Methodius, Trnava, Slovakia

**Keywords:** Ca_V_3.1, channel gating, T-type calcium channel, voltage regulation, voltage sensor

## Abstract

Contributions of voltage sensing S4 segments in domains I – IV of Ca_V_3.1 channel to channel activation were analyzed. Neutralization of the uppermost charge in individual S4 segments by exchange of arginine for cysteine was employed. Mutant channels with single exchange in domains I – IV, in two adjacent domains, and in all four domains were constructed and expressed in HEK 293 cells. Changes in maximal gating charge Q_max_ and the relation between Q_max_ and maximal conductance G_max_ were evaluated. Q_max_ was the most affected by single mutation in domain I and by double mutations in domains I + II and I + IV. The ratio G_max_/Q_max_ proportional to opening probability of the channel was significantly decreased by the mutation in domain III and increased by mutations in domains I and II. In channels containing double mutations G_max_/Q_max_ ratio increased significantly when the mutation in domain I was included. Mutations in domains II and III zeroed each other. Mutation in domain IV prevented the decrease caused by the mutation in domain III. Neither ion current nor gating current was observed when channels with quadruple mutations were expressed. Immunocytochemistry analysis did not reveal the presence of channel protein in the cell membrane. Likely, quadruple mutation results in a structural change that affects the channel’s trafficking mechanism. Altogether, S4 segments in domains I-IV of the Ca_V_3.1 channel unequally contribute to channel gating by voltage. We suggest the most important role of the voltage sensor in the domain I and lesser roles of voltage sensors in domains II and III.

## Introduction

Ca_V_3.1 channels together with Ca_V_3.2 and Ca_V_3.3 channels belong to the family of low voltage activated or T-type calcium channels. These channels are characterized by tiny macroscopic current with fast activation and inactivation kinetics and low voltage threshold for activation [1,2]. Because of negative activation threshold below −60 mV these channels can contribute to the initial phase of cell depolarization at the foot of an action potential. Ca_V_3 channels participate in physiological functions such as generation of low threshold spikes leading to neuronal burst firing, neuronal communication via neurotransmitter and hormone release and cell proliferation [3]. In spite of extensive research, structural determinants of negative activation threshold remain elusive.

Ca_V_3 channels share their protein structure with other voltage dependent calcium channels as well as with voltage dependent sodium channels. This structure consists of 4 domains (I-IV), each having 6 transmembrane segments (S1 – S6) with a large cytoplasmic C-terminus domain [4]. S1 – S4 segments in each domain are considered to form voltage sensors. S4 segments containing positively charged amino acid residues act as actual voltage sensing elements which move upon membrane depolarization from internal to external protein water crevice [5]. This transient charge movement mediated by small conformational changes of the S4 segments can be measured as so-called gating current. Movement of S4 segments represents an initial step in channel activation which is followed by pore opening and results in inward ion current [5,6].

In contrast to all other voltage dependent ion channels, the voltage dependence of gating current measured from Ca_V_3 channels is not negatively shifted relative to voltage dependence of ion currents [7]. It was hypothesized that the conductive pore of Ca_V_3 channels does not require activation of S4 segments in all four domains for its opening [7]. Contribution of individual channel domains to voltage dependent channel gating was investigated in several studies. Initially, structural determinants of pore opening were searched.

Generation of channel chimeras where individual Ca_V_3.1 domains were replaced by corresponding high voltage activated Ca_V_1.2 channel domains demonstrated that domains I, III, and IV, but not domain II, are critical for channel opening [8]. However, exchange of only the S4 segments did not provide evidence of active contribution to the difference in voltage dependent activation of these channel families [8]. Detailed studies on domain I by the same group pointed out its importance for channel activation and showed that the S5 and S6 region rather than voltage sensing S1-S4 were critical [9]. Swapping of S4 segments between Cav3.3 channels and Ca_V_1.2 channels demonstrated that IIS4 and, to a lesser degree IVS4, segments are crucial in determining the negative voltage threshold of Ca_V_3.3 channel activation [10].

Experimental approaches using exchange of one or several positively charged amino acid residues for uncharged amino acids in the IVS4 segment of the Ca_V_3.1 channel pointed to the role of domain IV in channel activation, but not in inactivation [11]. Effect of substitution of uppermost charged arginines by neutral cysteines in individual domains of the Ca_V_3.1 channel showed the role of S4 segments in domains I, II and III in channel activation [12]. The importance of the domain I in Ca_V_3 channel gating was underscored by several studies by the Perez-Reyes group describing a role of the putative gating brake in the intracellular loop connecting domains I and II in stabilizing the closed state of the channel [13,14].

Fewer studies addressed the role of activation of individual voltage sensors in Ca_V_3 channel activation. The lack of an effect of charge removal in the IVS4 segment of the Ca_V_3.1 channel on voltage dependence of gating current was demonstrated [15]. Removal of the gating brake in the I-II loop of Ca_V_3.1 and Ca_V_3.3 channels shifted voltage dependences of both gating and ion currents towards even more negative voltages suggesting a prominent role of domain I in Ca_V_3 channel activation [16,17].

In this study we addressed contribution of voltage sensors in individual domains of the Ca_V_3.1 channel to channel activation by neutralization of uppermost charge in individual S4 segments. We measured maximal charge movement from four single mutants (R180C – IS4, R834C – IIS4, R1379C – IIIS4, R1717C – IVS4), four double mutants (R180C+ R834C – I+ IIS4, R834C+ R1379C – II+ IIIS4, R1379C+ R1717C – III+ IVS4, R180C + R1717C – I+ IVS4) and one quadruple mutant (R180C+ R834C+ R1379C+ R1717C – I+ II+ III+ IVS4).

## Material and methods

### Mutagenesis and cell transfection

The mouse brain T-type Ca_V_3.1 calcium channel (accession number AJ012569) [18] was used for construction of mutants. cDNA for the channel was cloned into pEGFP N-1 plasmid (Clontech, Mountain View, CA, USA) with the stop codon removed, so that EGFP protein was fused to a carboxy terminus of the channel protein and enabled visual detection of successfully transfected cells. Mutations were introduced into S4 segments in individual channel domains using PCR-based methods [12]. Uppermost arginines in individual S4 segments were replaced by cysteines (R180C, R834C, R1379C, and R1717C). Series of four single mutants with a replacement in the single domain, four double mutants with replacements in two neighboring domains, and one quadruple mutant with replacement in all four channel domains were constructed. Detailed procedures were described previously [12].

cDNAs for wild-type and mutated Cav3.1 channels in pEGFP N-1 plasmids were transiently expressed in HEK 293 cells (DSMZ, Deutsche Sammlung von Mikroorganismen und Zellkulturen GmbH, German Collection of Microorganisms and Cell Cultures). Cells were grown in MEM with Earle’s salts, supplemented with 10% fetal calf serum and 100 U/ml penicillin-streptomycin in an atmosphere of 5% CO_2_ and 95% air at 37° C. Transfection of cells was done using the jetPRIME transfection reagent (Polyplus-transfection S.A., NY, USA) by the reverse transfection method where cDNA-reagent mixture is added to culture medium before adding of adherent cells. After 24 h transfected cells were split, seeded on cover glasses coated with poly-L-lysine and used for electrophysiological experiments 24 h later.

### Electrophysiology recordings and data analysis

Whole-cell calcium currents were measured using an EPC-10 patch clamp amplifier (HEKA Electronic, Lambrecht, Germany). Patch pipettes were made from borosilicate glass (Sutter Instrument, Novato CA, USA). When filled with intracellular solution, input resistance ranged between 1.8 and 2.1 MΩ. Cell capacitance and series resistance were monitored after membrane rapture and online compensated up to 70% by built-in circuits of the EPC-10 amplifier. Cell capacitance ranged between 9 and 34 pF. Only cells with series resistance less than 5 MΩ were taken into experiment. HEKA Patchmaster v90.2 was used to record raw data which were subsequently analyzed offline by HEKA Fitmaster v2x73.1 and OriginPro 2015 software. Extracellular solution contained (in mM): CsCl 95; TEACl (tetraethylamonium chloride) 40; BaCl_2_ 5; MgCl_2_ 1; HEPES (4-(2-hydroxyethal)-1-piperazineethanesulfonic acid) 10; glucose 10; pH 7.4 (CsOH) and intracellular solution contained of (in mM): CH_3_SO_3_Cs 130; Na-ATP 5, TEACl 10; HEPES 10; EGTA 10; MgCl_2_ 5; pH 7.4 (CsOH). Osmolarity of experimental solutions was measured by Osmomat 030 (Gonotec, Germany). Measured osmolarity of the intracellular solution was approximately 300 mOsmol/l and corresponding extracellular solution osmolarity was set to be 2–3 mOsm/l lower than the osmolarity of intracellular solution by adding sucrose.

Holding potential (HP) in all experiments was set to −100 mV. After stabilizing the whole cell configuration, series of 20 ms long depolarizing pulses from HP to potentials between −100 mV and + 70 mV with 10 mV increment were applied to determine current-voltage relation (IV) of ion current. The voltage-dependence of the peak Ba^2+^ current was fitted with the modified Boltzmann-Ohm equation:

(1) $$I\left(V\right)=G_{max}\frac{\left(V-V_{rev}\right)}{1+e^{\frac{\left(V_{0.5}-\;V\right)}{dV}}}$$

with *I*(*V*) representing the peak current amplitude measured at the depolarization potential *V, G*_max_ the maximum conductance, *V*_rev_ the reversal potential, *V*_0.5_ the half activation potential, and *dV* the slope factor.

Actual reversal potential for each examined cell was determined from a series of depolarizing pulses to amplitudes between + 40 and + 60 mV with an increment of + 2 mV. ON-gating currents Q_ON_ were measured by a set of five 20-ms long depolarizing pulses to a voltage corresponding to the value of reversal potential. Capacity transients and linear leak component were subtracted using – P/8 procedure. Recorded gating current traces were averaged. Resulting traces were used for calculation of total charge transferred at the beginning of the depolarizing pulses by integrating the area below the trace. The time course of gating current integral was used to determine 10–90% rise time of gating charge for each cell.

### Immunocytochemistry

To verify the presence of Ca_V_3.1 quadruple mutant protein in transfected cells we performed an immunofluorescence assay. HEK 293 cells expressing either the Ca_V_3.1 WT or the Ca_V_3.1 quadruple mutant seeded on coverslips were fixed with 4% formaldehyde for 10 min at RT, permeabilized in 0.1% Triton X solution and washed with 1% BSA and 0.1% Tween 20 in PBS for 1 h. Primary antibody (rabbit anti-Ca_V_3.1, AB5491, Chemicon International) was diluted 1:200 in PBS with 1% BSA and applied to cells overnight at 4°C. Next day, cells were incubated with secondary antibody anti-rabbit STAR-635P (Thermofisher) at a final dilution of 1: 1000 in dark at RT. Finally, coverslips with cells were mounted using Vectashield containing DAPI (Vector Labs) and taken for confocal microscopy.

### Confocal microscopy

Cellular distributions of Ca_V_3.1 wild-type (WT) channel and its quadruple mutant were examined using confocal microscopy. HEK 293 cells were transfected with cDNA for either Ca_V_3.1 WT or Ca_V_3.1 quadruple mutant using the method described above. Cells grown on coverslips were placed into a microscope chamber. Medium was changed for PBS and subsequently FM4-64 (Thermofisher) fluorescent dye (4 µM in PBS) was added directly to the chamber with cells.

Photomicrographs were taken with a Leica TCS SP8 STED 3X with HC PL APO CS2 63x/1.40 oil objective. Confocal aperture was set on 1 AU. Cav3.1 channels conjugated with EGFP were visualized with excitation 488 nm and fluorescence emission window of 498–578 nm. The outer leaflet of cell lipid membrane labeled by FM4-64 was visualized with 500 nm excitation whereas emission was detected between 650–764 nm. All images were scanned in x-y mode, with optimized pixel size 30–60 nm.

### Statistical analysis

Statistical analysis was performed using GraphPad InStat v 3.10 (GraphPad Software Inc.). All data are presented as mean ± S.E.M for *n* recorded cells. Individual data sets were checked for normal distribution. When one of the data sets did not pass the normality test, significance of difference between mutant groups was tested by Kruskal-Wallis test of nonparametric ANOVA followed by Dunn’s multiple comparisons test. Otherwise one-way ANOVA with Dunnett multiple comparisons test was used.

## Results

**Figure 1.**
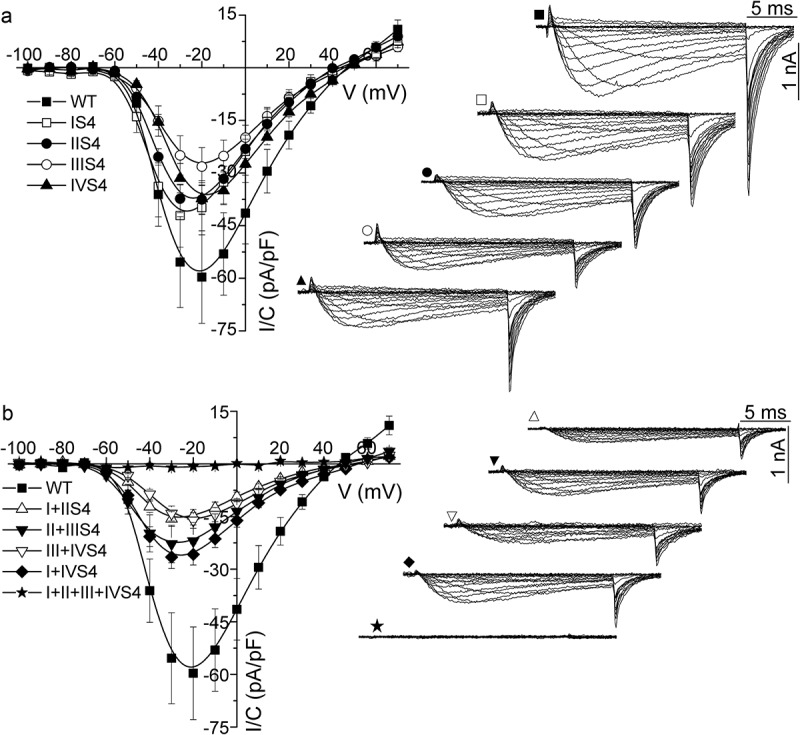
Effect of neutralization of uppermost arginine in S4 segments on ion currents through expressed Ca_V_3.1 calcium channel. (a), Averaged current-voltage relations from cells expressing WT channels and constructs with single arginine substitution in S4 segments of domains I – IV, as indicated. Representative examples of current recordings are presented in the right part of the panel. (b), Averaged current-voltage relations from cells expressing WT channels and constructs with double (I+ II, II+ III, III+ IV, I+ IV) and quadruple (I+ II+ III+ IV) arginine substitutions in specified domains, as indicated. Examples of current traces are presented on the right. Solid lines represent B-spline connectors of experimental points. Numbers of cells tested are summarized in Table 1.

To assess the effect of charge neutralization in voltage sensors on channel activation we evaluated the relation between ion current and gating current in each construct. First, we measured current-voltage relations (IVs). Averaged IV relations measured from wild type (WT) channel and channels bearing single, double, or quadruple mutation are presented in Figure 1. It is apparent that each single (Figure 1(a)) or double (Figure 1(b)) mutation resulted in decreased current density. No ion current was observed in cells transfected with the quadruple mutant (n = 51, Figure 1(b)).

For quantitative comparison data for each individual IV relation were fitted by a Boltzmann-Ohm Equation (1). Averaged fitting parameters are summarized in Table 1. Each single mutation caused a decrease in maximal conductance G_max_, but this decrease was significant only when a charge was removed in the IIIS4 segment. This mutation and mutation in IVS4 also significantly increased the slope of IV relations. All double mutants significantly decreased maximal conductance compared to the WT channel (Table 1). Further, all but the substitutions in IIIS4 significantly shifted the voltage dependence of current activation towards more negative voltages and all except IS4 mutations significantly increased slope of voltage dependence of current activation (Table 1).

**Table 1.** Comparison of activation parameters of WT and mutant Ca_V_3.1 channels.

	*N*	G_max_ (pS)	dV (mV)	V_0.5_ (mV)
WT	17	17.7 ± 2.1	−5.5 ± 0.3	−36.2 ± 1.7
IS4	16	12.6 ± 2.7	−5.7 ± 0.2	−41.1 ± 0.6*
IIS4	20	12.9 ± 1.5	−5.6 ± 0.2	−40.4 ± 0.6
IIIS4	16	9.4 ± 1.1**	−6.9 ± 0.2**	−34.2 ± 0.8
IVS4	13	13.0 ± 1.5	−6.6 ± 0.1**	−33.3 ± 0.5
I+ IIS4	10	4.6 ± 0.7***	−6.0 ± 0.2	−42.5 ± 0.3**
II+ IIIS4	16	7.4 ± 1.5**	−7.1 ± 0.3**	−41.8 ± 1.0**
III+ IVS4	12	7.3 ± 1.8**	−7.6 ± 0.3**	−34.7 ± 0.7
I+ IVS4	16	7.1 ± 0.7*	−6.4 ± 0.2*	−41.5 ± 0.6**

The maximal conductance G_max_, the steepness factor dV, and the half activation potential V_0.5_ were calculated by fitting IV relation data with the modified Boltzmann Equation (1). *N* is the number of analyzed cells. Values are presented as mean ± SEM. All data sets were checked for Gaussian distribution. Statistical significance was tested by Dunnett multiple comparison test of ANOVA. Data sets that did not follow Gaussian distribution were tested by Kruskal-Wallis test of nonparametric ANOVA followed by Dunn’s multiple comparison test. * *p* < 0.05, ** < 0.01, *** < 0.001.

**Figure 2.**
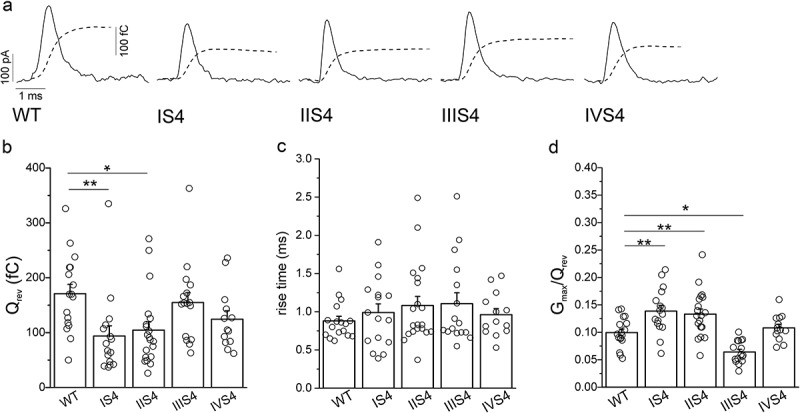
Effect of arginine neutralization in single domains of the Ca_V_3.1 calcium channel on gating current. (a), Representative gating current traces measured from cells expressing WT channels and individual single S4 mutants. Currents were recorded at the reversal potential of each cell. Overlapping dashed lines represent corresponding time courses of current integrals. (b), Averaged charge transfer (Q_rev_) values calculated as integral of the area under the gating current trace for cells expressing WT and single domain S4 mutant Ca_V_3.1 channels. (c), Averaged 10 −90 % rise time values of gating current integrals determined for WT channels and single S4 mutants. (d), Averaged G_max_/Q_rev_ values for WT channels and single S4 mutants. Data for individual cells analyzed in each group are shown as circles in bar diagrams (b, c, d). *, *p* < 0.05; **, *p* < 0.01.

**Figure 3.**
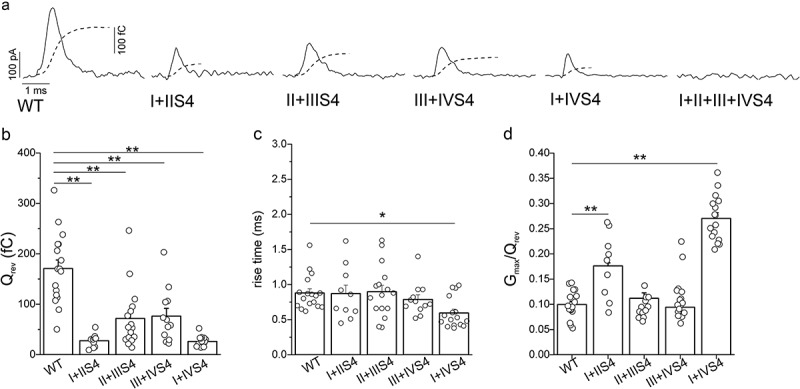
Effect of arginine neutralization in two neighboring domains of the Ca_V_3.1 calcium channel on gating current. (a), Representative gating current traces recorded from cells expressing WT channels, double, and quadruple mutants. Currents were recorded at the reversal potential of each cell. Overlapping dashed lines represent corresponding time courses of current integrals. (b), Averaged charge transfer (Q_rev_) values calculated as integral of the area under the gating current trace for cells expressing WT and double mutants. (c), Averaged 10 −90 % rise time values of gating current integrals determined for WT channels and double mutants. (d), Averaged G_max_/Q_rev_ values for WT channels and double S4 mutants. Data for individual cells analyzed in each group are shown as circles in bar diagrams (b, c, d). *, *p* < 0.05; **, *p* < 0.01.

For evaluation of voltage sensor activation we measured gating current at the reversal potential for each tested cell. At the reversal potential ion current is zero and gating current can be measured without blocking ion current [19]. Total ON-charge transferred during activation of channel voltage sensors evaluated as an integral of area under the gating current trace was significantly decreased by single mutations in segments IS4 and IIS4 (Figure 2(a,b)). Kinetics of voltage sensors movement were evaluated as a 10–90% rise time of current integral (Figures 2(a) and 3(a)). Single mutation did not affect these kinetics (Figure 2(c)). Ratio of maximal conductance and maximal ON-charge for each cell characterizes the efficiency of coupling between voltage sensor activation and opening of the channel pore. Mutations in IS4 and IIS4 significantly enhanced this coupling, while mutation in IIIS4 significantly attenuated it.

All double mutations significantly decreased ON-charge measured at the reversal potential (Figure 3(a,b)). Kinetics of activation of voltage sensors was significantly accelerated by mutations in segments IS4+ IVS4 (Figure 3(c)). Coupling between voltage sensor activation and pore opening was significantly enhanced by mutations in IS4+ IIS4 and IS4+ IVS4 (Figure 3(d)). No gating current was observed in cells transfected with the quadruple mutant (Figure 3(a)).

**Figure 4.**
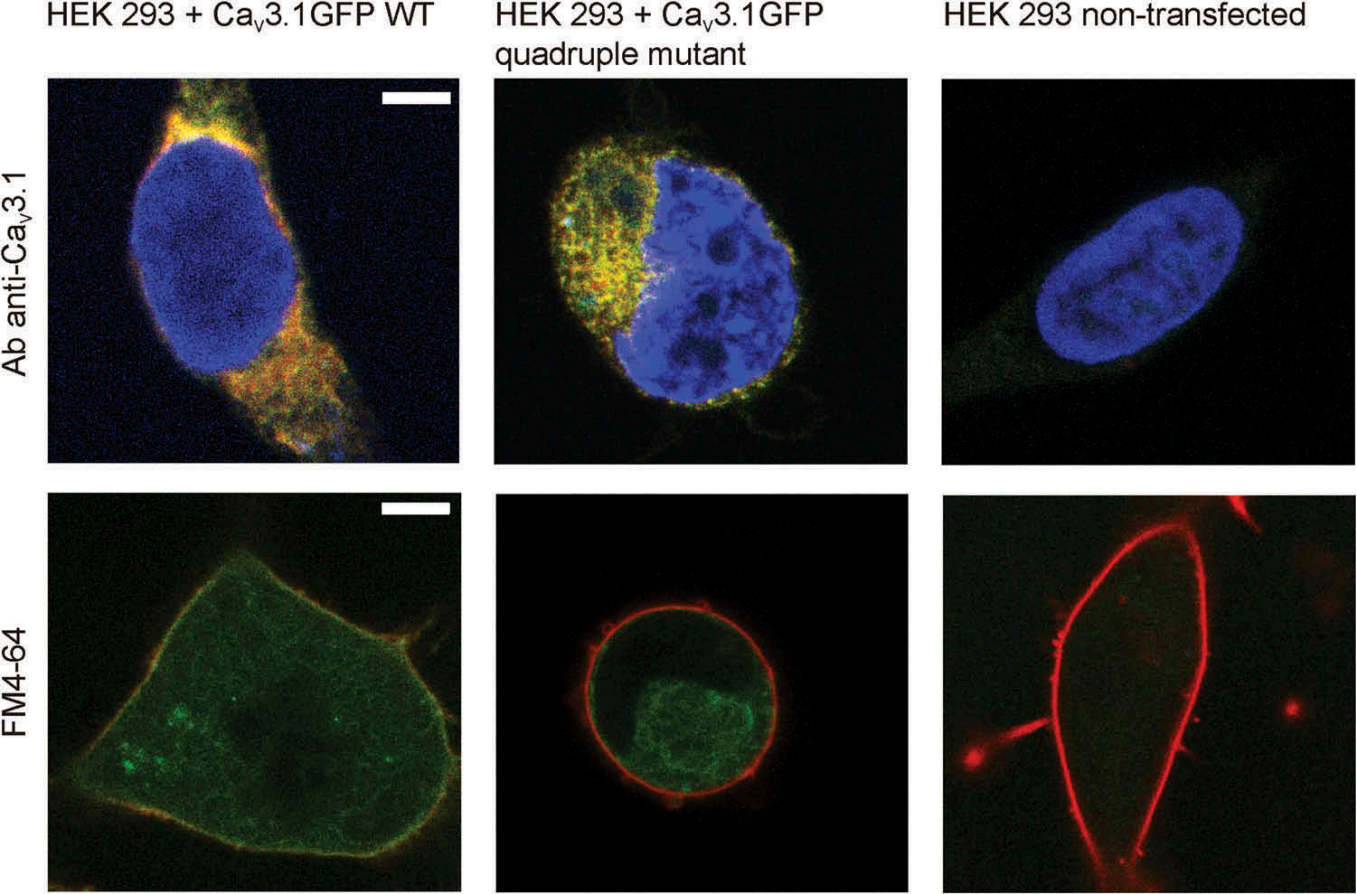
Expression of Ca_V_3.1 protein with quadruple mutation. The upper row shows representative immunofluorescent images of HEK 293 cells expressing Ca_V_3.1 WT channels, Ca_V_3.1 channels with quadruple mutations, and non-transfected HEK 293 cells with Ca_V_3.1 channels conjugated with EGFP (green). Cells were labeled with Ca_V_3.1 channel antibody (red) 72 h after transfection. Merged images show analogous expression pattern for both WT and mutant channels (yellow) and verify the intracellular distribution. The cell nucleus was stained using DAPI (blue). The lower row shows representative confocal images of HEK 293 cells expressing Ca_V_3.1 WT channels, Ca_V_3.1 channels with quadruple mutations, and non-transfected HEK 293 cells 72 h after transfection. Ca_V_3.1 channels were GFP-tagged (green) and cell membrane was FM4-64 stained (red). WT channels are localized within the lipid membrane (yellow signal, left panel), whereas the localization of Ca_V_3.1 channels with quadruple mutations (middle panel) within cell membrane is not identifiable. Scale bar represents 5 µm.

Complete loss of function in the quadruple mutant, i.e., loss of both gating and ion current, could be a result of a major defect in either channel gating or channel trafficking. To identify distribution of channel protein in cells we performed immunocytochemistry and fluorescent labeling (Figure 4). Immunocytochemistry labeling of cells transfected with Ca_V_3.1 WT or Ca_V_3.1 quadruple mutant using a Ca_V_3.1 antibody confirmed the suitability of the GFP-tag for determining the expression of calcium channel protein in cells. No differences in expression pattern and channel protein distribution were observed (Figure 4, upper row). Subsequent confocal micrographs of cells expressing GFP-tagged Ca_V_3.1 WT or Ca_V_3.1 quadruple mutant channels stained with FM4-64 cell membrane fluorescent dye showed the presence of both channel proteins throughout the cytoplasm. Co-localization of channel protein within cell membrane is evident in Ca_V_3.1 WT cells, but in cells expressing the quadruple mutant it is not clearly distinguishable (Figure 4, lower row, middle panel). These data suggest that quadruple mutant channel protein is expressed in cells but its trafficking to cell membrane may be defective.

## Discussion

In this study we analyzed contributions of S4 segments in domains I – IV of the Ca_V_3.1 channel to voltage dependent channel activation. Neutralization of the uppermost charge in individual voltage sensing segments by exchange of arginine for cysteine was employed to alter their function. The most prominent effect on channel conductance was caused by single mutation in domain III or by double mutation in domains I and II. Qualitatively similar changes were reported previously [12]. Different compositions of bath and pipette solutions do not allow us to compare previously reported values to those from our current study. Solutions used in this work were optimized for observation of gating current without necessity to block ion current. Properties of ion current through Ca_V_3.1 channels, e.g., maximal slope conductance, reversal potential, and shape of outward current markedly depend on the type of monovalent ions [20,21].

The main aim of this work was to elucidate contributions of individual voltage sensing S4 segments to channel activation. We concentrated on measurement of gating current at the ion current reversal potential of each investigated cell. Measurement of complete voltage dependence of gating current requires sufficiently high channel expression [7] and especially for double mutants it would not be possible to resolve gating currents at depolarization voltages above the activation threshold. By the definition, at the reversal potential inward current is equal to zero and does not superimpose onto the ON-gating current. The measured gating charge Q_rev_ can be considered to be closely approximate the maximal gating charge Q_max_ [13,19,22].

Charge movement measured at the reversal potential was most affected by single mutation in domain I and by double mutations including the same mutation (i.e., domains I + II and I + IV). Maximal conductance G_max_ depends on the number of channels expressed in particular cell and open probability of each channel. Maximal charge Q_max_ movement depends on a number of channels expressed in a particular cell and maximal charge transferred in each channel. The Ca_V_3.1 channel contains 22 charged amino acids in all four S4 segments [18]. In single mutants the total charge available for transfer in these segments is decreased by less than 5%, and in double mutants approximately by 9%. Therefore we can hypothesize that the total decrease in charge transfer could be attributed mostly to the decreased number of expressed channels (Table 2).

**Table 2.** Relative effects of mutations on maximal slope conductance and charge movement.

	G_max_		Q_rev_	
	decrease (% of control)	∑ of constructs	decrease (% of control)	∑ of constructs
IS4	−29		−45	
IIS4	−27		−38	
IIIS4	−47		−9	
IVS4	−27		−27	
I+ IIS4	−74	−56	−84	−83
II+ IIIS4	−58	−74	−58	−47
III+ IVS4	−59	−74	−55	−36
I+ IVS4	−60	−56	−85	−72

Relative decrease of G_max_ observed in single and double mutant channels was evaluated from averaged data summarized in the Table 1. ∑ of constructs represents simple summation of effects observed for those single mutations which are introduced in respective double mutant channel.

If the decrease in G_max_ would be solely due to the decreased number of channels in the cell membrane that are available for opening, this decrease should correlate to some extent with the decrease in Q_max_. Such rough correlation was observed for single mutation in domain IV and for double mutations in domains II + III and III + IV (Table 2). In all remaining mutations the ratio G_max_/Q_max_ was significantly altered. This ratio is proportional to the opening probability of the channel [19,22]. Our results indicate a decreased opening probability caused by the mutation in the domain III and increased opening probability by mutations in domains I and II. Results obtained for double mutations support such interpretations. When a mutation in domain I was included, G_max_/Q_max_ ratio increased significantly. Mutations in domains II and III zeroed each other. The mutation in domain IV itself did not affect channel opening, but prevented decrease caused by the mutation in domain III.

The lack of effect of the domain IV mutation on pore opening is in line with previous reports [11,15]. The importance of voltage sensors in domains I and II was suggested in several studies showing that removal of the so-called gating brake in the intracellular loop connecting domains I and II increases opening probability of both Ca_V_3.1 and Ca_V_3.3 channels [13,16,17] and shifts voltage dependencies of gating currents towards more negative potentials. Hence, S4 segments in proximity of the gating brake play an important role in channel activation, and molecular modeling suggests the voltage sensor in the domain I as a possible candidate [14,23]. This is in line with our observation that mutation in IS4 caused the most pronounced effect.

Mutations in domains I, II and III did affect pore opening of the channel by enhancing (I and II) or decreasing (III) the efficiency of coupling between voltage sensor activation and pore opening of the channel. However, the kinetics of charge movement were not altered by any of the investigated mutations, and therefore we hypothesize that speed of movement of individual S4 segments was not affected.

The lack of ion current through the quadruple mutant may be due to failure of coupling between voltage sensor activation and pore opening. In such a case the gating current from expressed channels should be measurable. In spite of great effort no gating current was detected. Therefore the most likely explanation is defective trafficking of channel protein to the cell membrane. Indeed, labeling of both channel protein and cell membrane did not show an overlap that was detectable at our resolution, suggesting that this channel was not properly inserted into cell membrane. Multiple structural elements of Ca_V_3 channels contribute to surface expression of the channel protein: the middle and distal part of the I-II loop [13,24], III-IV loop [25,26], amino terminal region [27], and external glycosylation sites [28,29]. None of these sites seems to be able directly interact with the uppermost arginines in S4 segments. However, positive charges in S4 segments are coordinated with negative charges in S2 and S3 segments [30]. We may speculate that disruption of such coordination in all four segments results in a structural change that negatively affects trafficking.

The role of voltage sensors in individual channel domains was analyzed in detail in Ca_V_1.2 channels [31]. These authors suggested that activation of voltage sensors in domains II and III is necessary for pore opening with a smaller contribution of the voltage sensor in domain I and no contribution of the voltage sensor in domain IV. Other groups pointed to a major role of voltage sensors in domain I, a lesser role of domain III and a minimal role of domains II and IV [32,33]. Here, we suggest that the voltage sensor in domain I is the most important for activation gating of the Ca_V_3.1 channel whereas the voltage sensors in domains II and III are less important.
